# Built and socioeconomic environment: associations with cancer-risk health behaviors among healthy emerging adults

**DOI:** 10.1007/s10865-026-00692-0

**Published:** 2026-06-19

**Authors:** Shannon Desbiens, Katie Darabos, Sean McHugh

**Affiliations:** 1 Rutgers School of Public Health, 683 Hoes Lane West, Piscataway, NJ 08854 USA; 2 Rutgers Cancer Institute, 195 Little Albany Street, New Brunswick, NJ 08901 USA

**Keywords:** Built environment, Socioeconomic environment, Health behaviors, Cancer prevention, Emerging adults

## Abstract

Health compromising behaviors such as tobacco use, binge drinking, physical inactivity, sedentary behavior, obesity, and inadequate sleep are associated with increased cancer risk. Emerging adults (age 18–29) may be especially vulnerable to engaging in health compromising behaviors due to the transitional nature and novel independence during this developmental period. The current study uses data from the 2022 Health Information Trends Survey Data Linkage Project to understand relationships between aspects of the built environment (i.e., the Food Environment and the Exercise Environment) and area-based socioeconomic status (i.e., Yost index) on health behavior profiles of healthy emerging adults in the United States. Latent class analysis identified two patterns of health behaviors, of which both were considerably unhealthy for engaging in different health behaviors pertinent to cancer risk. Binary logistic regression analyses revealed a significant association between emerging adults with lower access to exercise environments and a trend towards significance for those residing in median level socioeconomic status environment (SES) with emerging adults in health behavior Class 2. Analyses focused on identifying sociodemographic factors that increase cancer risk revealed that education level moderated the relationship between SES and health behavior class, and that education level and, separately, living in a rural area moderated the relationship between the exercise environment and health behavior class. Findings provide a greater understanding of emerging adults’ built and socioeconomic environments that present barriers to engaging in healthy lifestyles. The findings present opportunities for future research and evidence-based interventions to be conducted with consideration to the role of the built and socioeconomic environment in mitigating cancer risk among the emerging adult population.

## Introduction

Emerging adults, defined as those between the ages of 18–29, are in a critical developmental period in which lifelong health habits and lifestyles are beginning to take shape (Tanner & Arnett, 2016). This life stage encompasses several defining features and stressors that separate emerging adults from their adolescent and older young adult counterparts. This includes encountering new experiences and navigating changes (e.g., living away from parents for the first time) that are often associated with the transition into adult roles, such as establishing financial and residential independence, forming committed romantic relationships, and establishing a career (Arnett, 2000). Given these significant transitions, emerging adulthood is a critical period in the lifespan where onset of health compromising behaviors (e.g., smoking cigarettes, binge drinking, being obese) begins to develop (Daw et al., 2017). During this time period many emerging adults experiment with tobacco (commonly cigarettes and e-cigarettes) and alcohol consumption, often in unhealthy quantities, gain significant weight, and change their physical activity levels to become more sedentary (Kwan, 2012; Maggs et al., 2004; Nelson et al., 2008; Bourke et al., 2025). For example, 26.7% of emerging adults (18–25) engage in binge drinking according to National Institute on Alcohol Abuse and Alcoholism (2026). This behavior is often considered “normal” for emerging adults living in the U.S. as 21st birthdays occur, engaging in college pre-games/tailgates, and holiday breaks reinforce heavy and binge drinking episodes (Merrill & Carey, 2016). However, unhealthy behaviors (e.g., binge drinking) developed during emerging adulthood are often carried into adulthood (Patrick et al., 2024), shaping one’s lifestyle, affecting health, and in particular, may increase the risk of cancer (Berger, 2018; Hidayat et al., 2020).

Emerging adulthood may be an optimal time for establishing a primary prevention lens (i.e., reducing the risk of cancer before it begins) by targeting modifiable risk factors in individuals who are still healthy (Breiner et al., 2015; Holman et al., 2017; Vineis & Wild, 2014). The transitional nature of emerging adulthood, where novel health-compromising behaviors may be taking place, may present an opportunity to implement a primary prevention method. Primary prevention is a proactive effort to avoid disease incidence before disease onset (Albee & Ryan-Finn, 1993). Modifiable risk-factors that promote a primary prevention lens include abstaining from tobacco, avoiding obesity (> 30 BMI), maintaining physical activity (at least 150 min of moderate intensity physical activity), limiting sedentary time (no current federally published guidelines), abstaining from drinking (in particular, heavy drinking), and getting adequate sleep (i.e., having an intact circadian rhythm) among other recommendations (Arem & Loftfield, 2018; Clemente-Suarez et al., 2025). While cancer is often more common among older adults, early-onset cancer diagnoses (< 50 years old) are on the rise (Bleyer, 2023). The increasing incidence of early-onset cancer may reflect patterns of unhealthy lifestyles during young adulthood and may be exacerbated among certain sociodemographic groups (Yang et al., 2017).

Previous research has suggested that psychoeducation and increasing awareness is one promising approach to reducing engagement in cancer-risk health behaviors. This work is grounded in the Health Belief Model (i.e., knowledge about health outcomes can drive current health behaviors), which posits that awareness (e.g., perceived susceptibility and severity) and perceptions of cancer-risk associated behaviors (e.g., perceived benefits and barriers) are associated with adoption of health behaviors (e.g., action) (Luquis & Kensinger, 2019; Rosenstock, 1974). Yet, research has suggested that while emerging adults are aware of the risks (e.g., susceptibility, severity) of engaging in health-compromising behaviors (e.g., substance use), these behaviors are often central to their lifestyle (Merten et al., 2017). This warrants the necessity for a deeper exploration of external drivers of cancer-risk associated health behaviors among emerging adults such as the environment in which they live.

Research has suggested that cancer-related health risk behaviors in adulthood often originate from an earlier age (Ugai et al., 2022). This stability of health behaviors over time is suggested to occur due to two factors rooted in health lifestyle theory (Cockerham, 2005). One being life choices, which requires a sense of agency that is often developed during emerging adulthood. Here, emerging adults are evaluating and choosing a course of action; do I start smoking cigarettes? Second being life chances, or class circumstances, as well as aspects of their lived environment. Here, those living in high socioeconomic status areas and/or those with better area-based food/exercise environments may be more likely to engage in health-promoting behaviors.

Bronfenbrenner’s socioecological systems theory of human development (Bronfenbrenner, 1977) also describes this interaction, in which an individual’s development is influenced by their interactions with aspects of the environments that surround them. These theories (i.e., socioecological systems theory and health lifestyle theory) suggest that interventions that target health behaviors without addressing the underlying environment will be unsuccessful. Indeed, there is a growing body of literature demonstrating that the socioeconomic and built environment are important contributors to health behaviors (Boone-Heinonen et al., 2011; Cho & Park, 2017; Hobbs et al., 2022; Howell & Booth, 2022; Huang & Sparks, 2023; Wong et al., 2022).

The socioeconomic environment refers to the area-based socioeconomic composition of county-level residents. Research has suggested that lower SES individuals may be exposed to less activity-supportive environments and live in worse food environments (Lovasi et al., 2009; Sallis et al., 2012). Social and neighborhood economic vulnerabilities have additionally been associated with declines in adherence to cancer prevention behaviors (i.e., cancer screening) (Mayhand et al., 2021) and with poorer cancer prognosis (Chao et al., 2015; Ko et al., 2024; Rodriguez et al., 2024).

The built environment refers to the human-made design and layout of neighborhoods and its physical structure, including retailer mix, street quality and connectivity, sidewalks, and greenspace (Lawrence & Low, 1990). Several studies among the general population have suggested that access to certain aspects of the built environment (e.g., fitness centers, tobacco outlets, alcohol outlets) are associated with both health-promoting and health-compromising behaviors (e.g., physical activity, smoking, drinking) (Lake & Townshend, 2013; Lipperman-Kreda et al., 2020; West et al., 2010).

In addition, it is well documented that there are clear differences in health behaviors by sociodemographic factors (e.g., race-ethnicity, gender, education, income) (Segrin et al., 2023). However, disadvantaged groups are not always more likely to exhibit high risk in terms of health behaviors. For example, among emerging adults, rates of tobacco and alcohol use among African Americans and Hispanics are lower than White emerging adults, as these behaviors tend to begin at older ages for non-Hispanic white individuals (Evans-Polce et al., 2015; Park et al., 2018; White et al., 2004). One reason for that may be differences in the environments of emerging adults. It is possible that the relationship between environment characteristics and health behaviors vary by sociodemographic factors (Christie-Mizell, 2022). However, there remains a gap in our understanding of the role of subgroup differences within associations of the socioeconomic and built environment and the clustering of health behaviors, particularly for healthy emerging adults.

Together, this growing body of literature demonstrates that aspects of the built and socioeconomic environments are related to lifestyle behaviors such as physical activity, substance use, sleep, sedentary behavior, and being overweight/obese that are associated with cancer-risk (Bernstein et al., 2007; Howell & Booth, 2022; Molina-García et al., 2019; Parks et al., 2023; Travis et al., 2022). However, most of the research to date has focused on associations between the built and socioeconomic environment on individual health behaviors. Yet, health behaviors may cluster in unique ways and often co-occur (Daw et al., 2017; Lawrence et al., 2017). While previous research has contributed greatly to the understanding of engagement in health behaviors among emerging adults, to design effective interventions, research must move towards identifying distinct patterns of risk behaviors and how aspects of the built and socioeconomic environments are associated with these patterns. Thus, in the current study, latent class analysis was used to identify latent subclasses of emerging adults who display similar patterns of engagement in health behaviors. We then examined relationships between the built environment (food environment index, access to places for exercise) and socioeconomic environment [area-based socioeconomic status (SES)] on health behavior class. Additionally, we examined the moderating role of sociodemographic factors on the relationship between the built and socioeconomic environment on health behavior class. Based on previous research, we hypothesized that health-promoting and health-compromising behaviors would cluster together, forming a “healthier” class and an “unhealthier” class and that lower accessibility to resources for health (i.e., low food and/or exercise environment, low area-based SES) would be associated with a class pattern high in health-compromising behaviors.

## Methods

### Study sample

This study used data from the Health Information Trends Survey (HINTS). HINTS is a national cross-sectional survey administered by the National Cancer Institute to US adults aged 18 and older (Finney Rutten et al., 2020). Data were drawn from the HINTS Data Linkage Project 2022 (HDLP) which is a restricted-use, geocoded, combined dataset of HINTS (Cycle 6) (Hesse & Moser, 2024) data which includes contextual variables coded at different geographic units (i.e., county-level, census-tract). The HINTS Data Linkage Project links participant data with data with several external variables chosen from reliable sources including the US Department of Agriculture, the US Census, and the Agency for Healthcare Research and Quality. External variables fall into five different categories in which for the current study we focused on the county level aspects of social and economic factors (i.e., Yost index for area-based SES) and the built environment [i.e., food environment index, adequate access to places for physical activity (i.e., exercise environment index)]. Health behaviors were self-reported from participants. Data were filtered to only include emerging adults (aged 18–29) who had no self-reported history of cancer (*N* = 391). Of note, there is considerable overlap in the literature regarding the definition of emerging adulthood. We chose to use Arnett’s definition of 18–29 years of age, as it may broadly capture the most transitional parts of life (between adolescents and young adulthood; for example, residential change, career building, romantic partner establishment) (Arnett & Mitra, 2020; Barlett et al., 2020; Frech, 2014).

### Measures

*Covariates.* Covariates were identified by significant associations between sociodemographic variables and health behavior class (i.e., race/ethnicity, education, metropolitan living) and a priori (i.e., gender) given previous research suggesting gender differences in health behavior engagement (Kritsotakis et al., 2016; Olson et al., 2017). *Gender* was measured using the self-reported gender on the original birth certificate. Only male and female response options were retained in the analysis. *Race* was assessed using two measures asking about race and ethnicity. Responses were combined and recategorized into three categories: Hispanic (i.e., all races who identified with a Hispanic ethnicity), Non-Hispanic White, and Non-Hispanic Other (which included Black or African American, American Indian or Alaskan, Asian, Native Hawaiian or other Pacific Islander, and multiple races selected). *Education* was assessed by asking participants the highest grade or level of schooling that they have completed. Based on sample distributions, the variable was dichotomized into two categories: less than a college degree and college degree or higher. *Metropolitan living* was provided within the HINTS Data Linkage Project based on data from the USDA 2010 Primary Rural-Urban Community Area Code (U.S. Department of Agriculture, Economic Research Service, 2026). Based on sample distributions, the variable was dichotomized: living in a metropolitan/urban area and not living in a metropolitan area/rural (which included micropolitan, small town, and rural area codes).

*Socioeconomic environment.* The Yost Index (Yost et al., 2001) is a measure of area-based socioeconomic status (SES) provided in the HDLP pulled from the American Community Survey (ACS) 2018–2022 (U.S. Census Bureau, 2023). Composite Yost Index scores were derived using 7 variables: median household income, median home value, median gross rent, percentage of persons below 150% of the federal poverty level, education index, percentage of working-class persons, and unemployment rates (Yost et al., 2001). Scores represent the relative SES level of a given geographical area. For the present study, we utilized the stratified version of the variable, which placed participants into 1 of 5 quintiles based on their original composite score. Higher quintile grouping indicates the status of living in a higher SES county (i.e., more affluent).

*Built environment*. The built environment (BE) was assessed using county-level within state quartiles of the food and exercise environment from HDLP, which extracted built environment data from the County Health Rankings (University of Wisconsin Population Health Institute, 2022).

The *Food Environment* represents accessibility to healthy foods by considering the distance to a grocery store or supermarket and the inability to access healthy food because of transportation barriers and financial instability. These data are pulled from the USDA Food Environment Atlas and Map the Meal Gap from Feeding America to assemble statistics on food environment factors from 2019 to 2022 (County Health Rankings, 2025). The food environment index ranges from a scale of 0 (worst) to 10 (best). For the present study, we utilized the stratified version of the variable, which placed participants into 1 of 4 within-in-state quartiles based on the county food environment index.

The *Exercise Environment* represents the percentage of the population in proximity to places for physical activity such as sidewalks, parks, recreational facilities and gyms. Individuals were considered to have adequate access to physical activity if they resided in a census block within a half mile of a park; within one mile of a recreational facility in an urban area; or within three miles of a recreational facility in a rural area. To create the exercise environment index, multiple data files (e.g., USA Parks data, YMCA location data, Census data of urban areas, roads, and rivers) are combined in ArcGIS Pro from 2021 to 2022 (County Health Rankings, 2025). For the present study, we utilized the stratified version of the variable, which placed participants into 1 of 4 within-in-state quartiles based on the county exercise environment index.

*Health Behaviors.* We included six self-reported health behaviors that are pertinent to cancer risk from the HINTS Cycle 6 data (HINTS Data Linkage Project, 2022). Health behaviors were dichotomized for the purposes of our analyses (latent class analysis).

*Obesity* was defined using BMI, in which scores of 30 or more were categorized as obese, and scores of 18-29.9 (scores below 18 were excluded for being unhealthy) were considered non-obese in accordance with the National Health Institutes Body Mass Index (National Heart, Lung, and Blood Institute, 2022).

*Binge drinking* was assessed based on the question asking individuals if they have had 4 or more drinks (females) or 5 or more drinks (males) on one occasion in the past 30 days. Those that responded “yes” were categorized as an active binge drinker.

*Physical activity* was defined as having engaged in moderate physical activity for 150 min or more per week. Participants who reported less than 150 min of exercise were categorized as not meeting physical activity recommendations as per guidelines of the CDC (Centers for Disease Control and Prevention, 2023).

*Sedentary behavior* was defined by the time reported sitting on a typical day at home or work. Participants reporting time above the median (i.e., 7 h) were categorized as being sedentary according to the study sample, as there are no concrete guidelines for sedentary behavior (Arem & Loftfield, 2018) and rather limitations are encouraged (Bull et al., 2020).

*Inadequate sleep* was defined as not reaching at least 7 h of sleep on average during the past 7 days as per guidelines of the Joint Sleep Consensus of the American Academy of Sleep Medicine and Sleep Research Society (Watson et al., 2015).

*Tobacco use* was defined by selecting “Current” to cigarette smoking status or e-cigarette use status. Although cigarettes and e-cigarettes present different risk-factors for cancer, these samples were combined due to very low cigarette (5.4%) and e-cigarette (10%) active-use statuses. Other forms of tobacco use (e.g., smokeless tobacco or cigars) were not available in the data set.

### Statistical analyses

Latent class analysis (LCA) was used to identify distinct, latent classes based on response patterns in the dichotomous health behaviors defined above using Mplus Version 8.11 (Muthén & Muthén, User Guide 1977–2017). LCA was chosen as the current study aimed to identify clusters of health behaviors. LCA uses indicators that are all measured on the same scale, typically dichotomous indicators. Dichotomous indicators are often easier to interpret than continuous indicators and can facilitate the assessment of class homogeneity and separation (Masyn, 2013; Ulbricht et al., 2018). Dichotomizing health behaviors is sufficient to meaningfully represent the variable and address the research question. The best fitting class is identified by running a series of latent class analyses, first fitting a model that is constrained to one-class, and then re-running the model constrained to two-classes, three classes, etc., until the model fails to converge or model indices failed to suggest that the higher class was a better fit for the data. Fit indices for each class model were examined to determine the best fitting class. This included the Akaike Information Criteria (AIC) and the Bayesian Information Criteria (BIC). Low AIC and BIC values are generally deemed to be indicators of good fitting models (Lanza et al., 2015). In addition, the boot-strapped likelihood ratio test (BLRT) was used for model comparison. An insignificant BLRT p value (*p* > 0.05) indicates that the model with one fewer class is a better fit for the data (McLachlan & Peel, 2000; Nylund et al., 2007). Model entropy was also examined with values above 0.80, suggesting classes that are well separated and distinct (Celeux & Soromenho, 1996).

Descriptive statistics, crosstabs analyses, and bivariate correlations were conducted for key study variables. Associations between sociodemographic variables were examined as possible covariates. Binary logistic regression was conducted to examine associations between socioeconomic (Yost Index) and built environment (food environment index, exercise environment index) variables on health behavior class in one model controlled for relevant sociodemographic variables identified in the bivariate analyses. Given smaller sample sizes in quartiles, exercise environment index quartiles were recategorized into a binary variable (quartile 1 remained the same; quartiles 2–4 were collapsed together). The Nagelkerke *R*^2^ was used to report the effect size, which emulates properties similar to the *R*^2^ in a multiple regression (Walker & Smith, 2016).

To test possible moderation effects, we included sociodemographic variables identified as significantly associated with health behavior class in the bivariate analyses and those identified a priori (i.e., gender). Moderation models were tested via the PROCESS macro for SPSS with bootstrapped confidence intervals at 5000 resamples to test effects (Hayes, 2017; Taylor et al., 2008). In each regression model, relevant covariates were entered into the first block, Yost index, food environment index, and exercise index were entered separately into the second block, and the interaction term (health behavior class x sociodemographic factor) in the last block. Interaction terms were analyzed in accordance with methods outlined by Aiken and West (1991). Simple slopes analyses were used to determine significant interactions at one standard deviation above and below the mean.

## Results

### Descriptive and identification of covariates

Demographic and health behavior characteristics of the study sample are shown in Table 1. Participants ranged from 18 to 29 years old (*M*^age=^24.7, *SD* = 3.4), were predominantly female (63%) and identified as non-Hispanic White (43%), Hispanic (28.4%) or non-Hispanic other race (28.6%; Black, Asian, American Indian or Native Alaskan, Native Hawaiian or Pacific Islander, Mixed race, or another category). This racial and ethnic spread generally reflects the trends of proportions from the 2020 US Census data (Jones et al., 2021). A little less than half of the participants (44.5%) lived in a metropolitan area and 55.5% held a college degree or higher (e.g., graduate degree). Regarding cancer risk associated health behaviors, 25.6% of our sample was obese, 38.6% of our sample were active binge drinkers at the time of data collection, 59.1% did not meet weekly exercise recommendations (≥ 150 min per week), 51.2% were sedentary for 7 h a day or more, 33.8% got less than 7 h of sleep, and 12.8% were current tobacco users (i.e., cigarette or e-cigarette users).

**Table 1:** Class and full sample descriptives statistics

Variable	Health behavior profile		
	Class 1	Class 2	Full sample
Sample size	151 (38.6%)	240 (61.4%)	391 (100%)
Age *M(SD)*	25.2 (2.852)	24.3 (3.591)	24.6 (3.346)
Gender			
Male	51 (33.8%)	94 (39.2%)	145 (37.1%)
Female	100 (66.2%)	146 (60.8%)	246 (62.9%)
Race			
Hispanic	51 (33.8%)	60 (25%)	111 (28.4%)
Non-Hispanic White	72 (47.7%)	96 (40%)	168 (43%)
Non-Hispanic Other	28 (18.5%)	84 (35%)	112 (28.6%)
Metropolitan^a^			
Living in Metropolitan	75 (49.7%)	99 (41.3%)	174 (44.5%)
Living in non-metropolitan	76 (50.3%)	141 (58.8%)	217 (55.5%)
Education			
Less than a college degree	62 (41.1%)	124 (51.7%)	186 (47.6%)
College/Advanced degree	89 (58.9%)	116 (48.3%)	205 (52.4%)
Health behaviors			
BMI			
Not obese	110 (72.8%	181 (75.4%)	291 (74.4%)
Obese	41 (27.7%)	59 (24.6%)	100 (25.6%)
Binge drinker			
Non-binge drinker	0 (0%)	210 (91%)	210 (62.8%)
Active binge drinker	151 (100%)	30 (9%)	181 (54.2.6%)
Physical activity			
≥ 150 min	78 (51.7%)	82 (34.2%)	160 (40.9%)
< 150 min	73 (48.3%)	158 (65.8%)	231 (59.1%)
Sedentary^b^			
< 7 h	68 (45%)	116 (48.3%)	184 (47.1%)
≥ 7 h	81 (53.6%)	119 (49.6%)	200 (51.2%)
Sleep			
≥ 7 h	103 (68.2%)	156 (65%)	259 (66.2%)
< 7 h	48 (31.8%)	84 (35%)	132 (33.8%)
Cigarette/E-Cigarette use			
Never/Former	120 (79.5%)	221 (92.1%)	341 (87.2%)
Current	31 (20.5%)	19 (7.9%)	50 (12.8%)

^a^Living in a metropolitan area was defined by living in an urban area code: 1 (Metropolitan core), 2 (Metropolitan high commuting), or 3 (Metropolitan low commuting). All other categories (4–10) were coded as not living in a metropolitan area/rural. This dichotomized coding scheme is in accordance with the U.S. Department of Health and Human Services, Federal Office of Rural Health Policy (U.S. Department of Agriculture, Economic Research Service, 2025).

^b^Cases (*n* = 7) with missing data were included in the analysis for the sedentary behavior.

Chi-square analyses were conducted to test associations between sociodemographic variables and health behavior classes. Significant or approaching significance (*p* ≤ 0.10) associations of race/ethnicity (^*2*^ = 12.6, *p* = 0.002), education (^*2*^ = 4.2, *p* = 0.04), and metropolitan (^*2*^ = 2.7, *p* = 0.10) area were controlled for in regression models. While there were no significant associations for gender (^*2*^ = 1.2, *p* = 0.28), gender was controlled for a priori given associations in the emerging adulthood health behavior literature (Kritsotakis et al., 2016; Olson et al., 2017).

### Latent class analysis

**Fig. 1:**
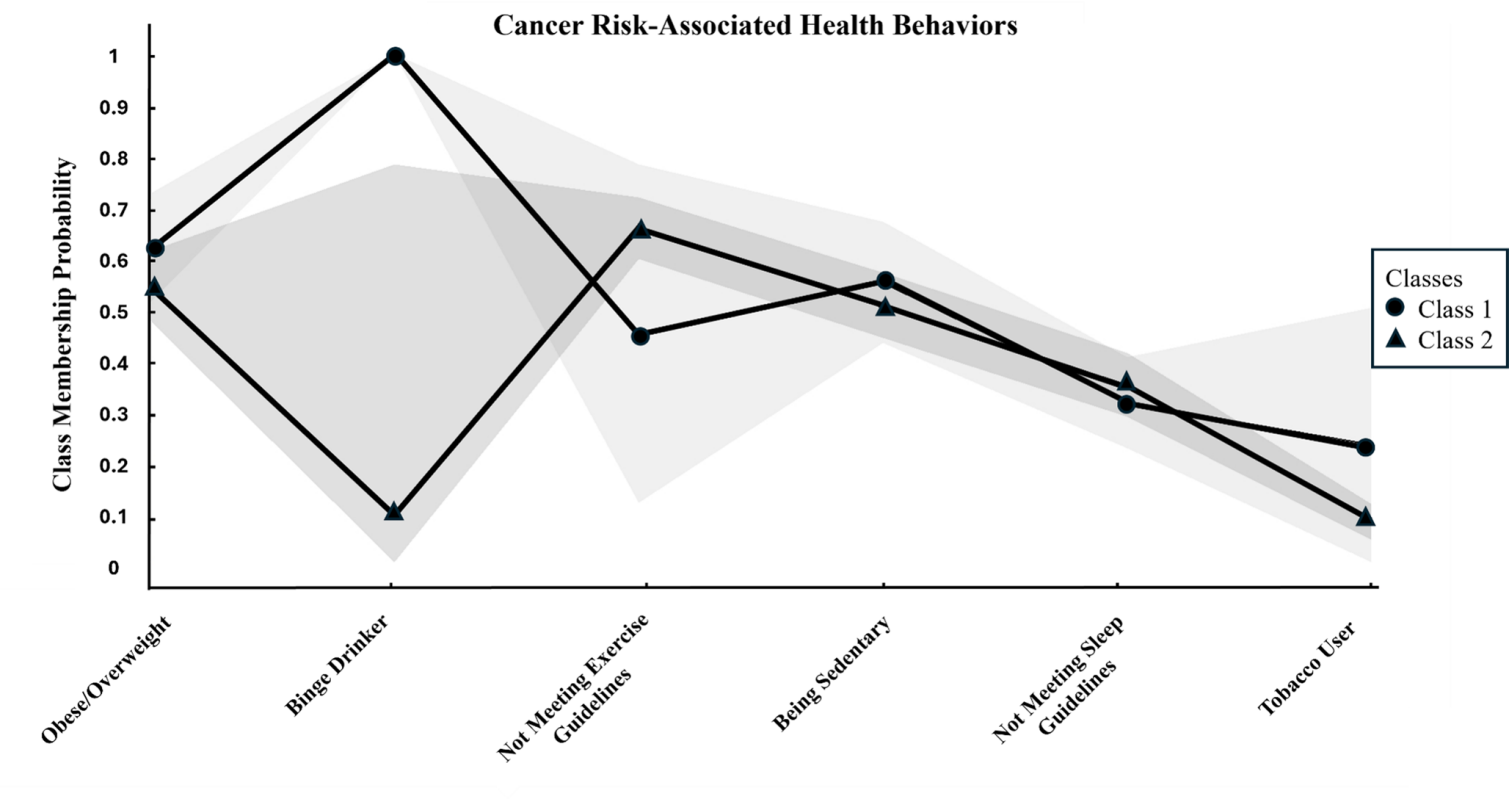
Latent class analysis health behavior proportions with plotted confidence intervals (95%)

Health behavior classes were obtained by adding classes and comparing the model fit until the tested model failed to converge or did not provide a better fit for the data. The best model fit was retained with a 2-class model (AIC = 2914.934, BIC = 2966.528). The 3-class model produced a significant BLRT and a larger BIC (2997.053), thus the model for two classes was retained and used in subsequent analyses (entropy value = 0.80). Figure 1 displays a plot of the two-class solution where each health behavior is presented on the x-axis, and the item-class probabilities are plotted on the y-axis. Corresponding confidence intervals are also plotted. Table 1 describes the demographic characteristics of the latent classes. Each participant had a probability score for placement into each class. Class 1 described 38.6% (*n* = 151) of the sample. Individuals in this class demonstrated a 27.7% (*n* = 41) likelihood of being obese, 100% (*n* = 151) likelihood of being an active binge drinker, 48.3% (*n* = 73) likelihood of not meeting exercise guidelines, 53.6% (*n* = 81) likelihood of being more sedentary than the rest of the sample, 31.8% (*n* = 48) likelihood of not getting enough sleep, and a 20.5% (*n* = 31) likelihood of being a current smoker/ e-cigarette user. Class 2 described 61.4% (*n* = 240) of the sample. Individuals in this class demonstrate a 24.6% (*n* = 59) likelihood of being obese, 9% (*n* = 30) likelihood of being an active binge drinker, 65.8% (*n* = 158) likelihood of not meeting exercise guidelines, 49.6% (*n* = 119) likelihood of being more sedentary than the rest of the sample, 35% (*n* = 84) likelihood of not getting enough sleep, and a 7.9% (*n* = 19) likelihood of being a current tobacco user (i.e., cigarette or e-cigarette user).

### Binary logistic regression model testing

Results from the logistic regression assessing socioeconomic (Yost Index) and built environment (food environment, exercise environment) factors on health behavior class are presented in Table 2. Significant covariates in the model include being non-Hispanic Other Race (OR = 3.84, 95% CI: 2.04–7.23) and having a college degree or higher (OR = 0.58, 95% CI: 0.36–0.94). For the built and socioeconomic variables, being in the lowest level exercise environment was associated with being in Class 2 (OR = 2.91, 95% CI: 1.34–6.32). Being in a median level socioeconomic status environment approached significance with being associated with Class 2 (OR = 2.35, 95% CI: 0.93-5.94). The Food environment was not significantly associated with the health behavior classes.

**Table 2:** Binary logistic regression for health behavior profile outcomes (*n* = 391)

Variable	R^2b^	OR (CI)^a^	*p* ^c^
Gender			
Male		Referent	
Female		0.75 (0.47–1.19)	0.214
Race/Ethnicity			
Hispanic		Referent	
Non-Hispanic White		1.54 (0.88–2.69)	0.131
Non-Hispanic all others		3.84 (2.04–7.23)	< 0.001**
Education			
Less than a college degree		Referent	
College degree/Advanced degree		0.58 (0.36–0.94)	0.027**
Metropolitan			
Living in metropolitan area (urban)		Referent	
Living anywhere else (rural)		1.26 (0.75–2.12)	0.390
Yost quintiles			
Quintile 1		Referent	
Quintile 2		1.78 (0.79–4.01)	0.163
Quintile 3		2.35 (0.93–5.94)	0.071*
Quintile 4		1.66 (0.68–4.02)	0.263)
Quintile 5		1.07 (0.42–2.76)	0.884
Food environment access quartiles			
Quartile 1		Referent	
Quartile 2		1.29 (0.71–2.35	0.407
Quartile 3		2.28 (0.81–6.43)	0.119
Quartile 4		1.82 (0.58–5.75)	0.307
Exercise environment access quartiles			
Quartile 1		Referent	
Quartile 2		2.91 (1.34–6.32)	0.007**
Quartile 3		3.14 (0.60-16.31)	0.174
Quartile 4		0.94 (0.07–12.43)	0.963

^a^Regression coefficients are reflective of the values with all variables entered in the model at the end of block 2.

^b^Only significant environment variables from the logistic regression were entered into the moderation models. The nagelkerke pseudo R^2^ scores are reported for the model fit.

^c^*p* < 0.05** *p* < 0.1*

### Moderation model testing

**Fig. 2:**
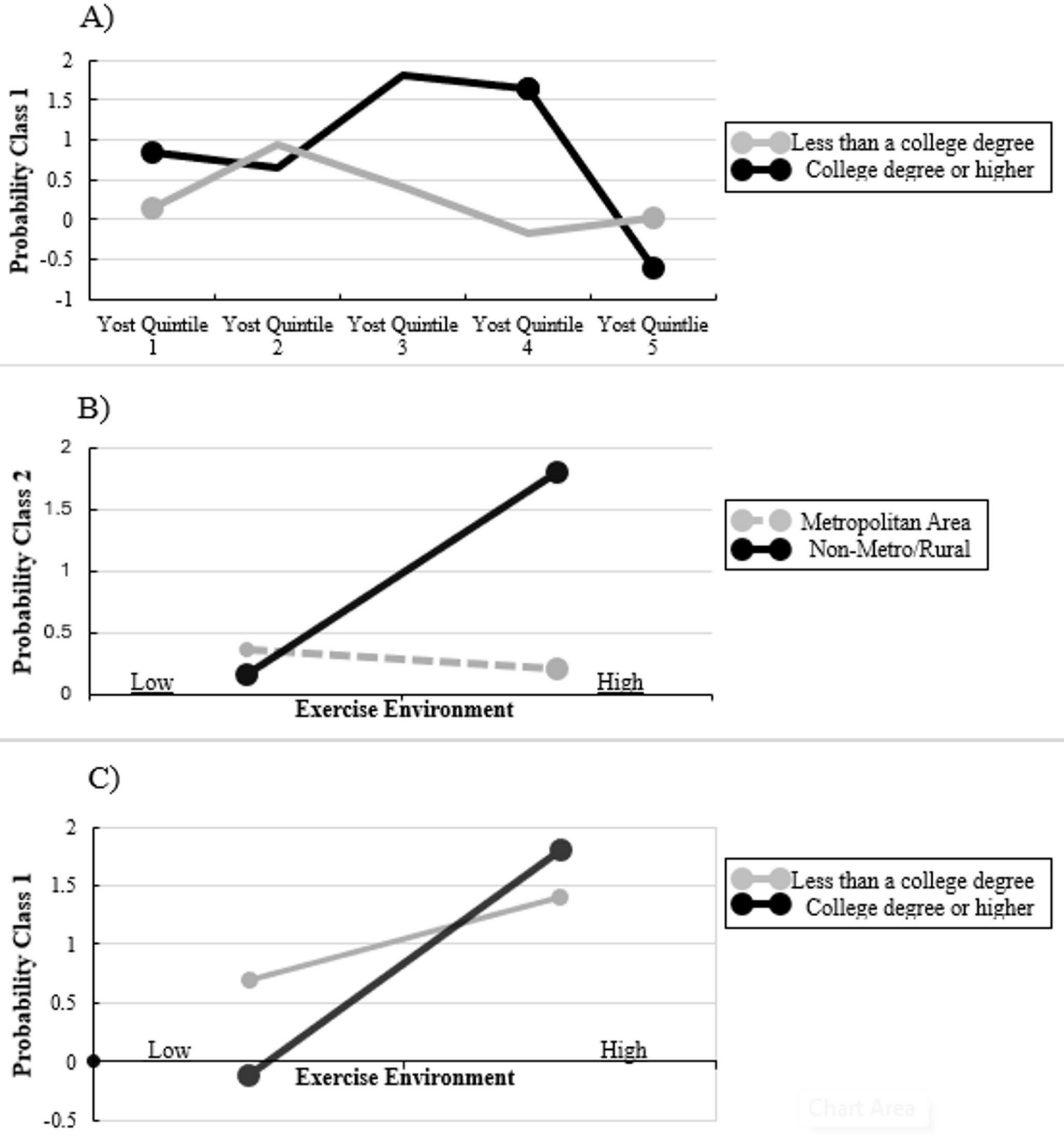
Simple slopes of the moderated sociodemographic variables on the environment. *Note*: Dashed lines represent insignificant slopes

Moderation models were run separately testing the moderating effects of race/ethnicity, gender, education, and metropolitan living on the association between socioeconomic (Yost Index) and built environment variables [food environment index (quartile), exercise environment index (binary)] and health behavior class. Simple slopes of significant interactions figures are presented in Fig. 2.

There was a significant interaction of education on the association between the Yost index and health behavior class (2 = 15.69, *p* = 0.004). Specifically, having less than a college degree was associated with being in Class 1 for those in the highest county level socioeconomic group (*b*= -1.43, *p* = 0.004). No other moderators tested were significant on the association between the Yost Index and health behavior class. [Simple Slope Fig. 2. Graph A].

There was a significant interaction of metropolitan living on the association between the Exercise Environment and health behavior class (2 = 4.38, *p* = 0.036). Specifically, those living in more rural areas and living in a higher exercise environment were associated with being in Class 2 (*b* = 1.63, *p <* 0.001) [Simple Slope in Fig. 2. Graph B]. Additionally, there was a trend towards education moderating the relationship between the Exercise Environment and health behavior class (2 = 3.64, *p* = 0.056). This indicates that having a college degree and living in higher exercise environments is moderately associated with being in Class 2 (*b* = 1.92, *p <* 0.001) [Simple Slope in Fig. 2. Graph C]. No other moderators tested were significant with the association between the Exercise environment and health behavior class.

There were no significant moderating interactions of sociodemographic variables between the Food Environment and health behavior classes.

## Discussion

The current study examined associations between the socioeconomic and built environment on health behavior profiles in a nationally representative sample of healthy U.S. emerging adults. While we hypothesized that healthy and unhealthy behaviors would cluster together, forming a “healthier” and an “unhealthier” class, contrary to our expectations, the best fitting model did not yield a clear healthy and unhealthy group, rather, both classes exhibited a high likelihood of engaging in health-compromising behaviors (i.e., binge drinking, physical inactivity, obesity) notably associated with increased cancer risk in the broader literature (Arem & Loftfield, 2018).

Class 1 was defined by binge drinking (100%) and a lower likelihood of being inactive as compared to Class 2, who had a very low chance of binge drinking (9%) and was more likely to be inactive relative to Class 1. Also, interestingly, Class 1 (less inactive) had a higher odds of being sedentary than Class 2, meaning that even though they are more likely to workout, they are less active throughout the day. Not meeting sleep recommendations was insignificant for both classes, as was tobacco use. Given our sample having remarkably low rates of tobacco use (15.4%), these ratios are unsurprising. However, it is notable that Class 1 is more likely to smoke cigarettes or use e-cigarettes, as well as binge drink and exercise. Here, we may be seeing compensatory behaviors through exercising within Class 1. Further research that observes substance user’s profiled behaviors may be more insightful for targeting this archetype of behavior and replacing substance use with healthier coping mechanisms. The fact that many emerging adults have knowledge on cancer risk associated health behaviors, yet still engage in them (Merten et al., 2017), suggests that external factors likely play a role in the adoption of health behaviors (health lifestyle theory and ecological systems theory). Targeting these populations through an ecological lens may yield progress in mitigating cancer risk-associated health behaviors in this class of health behavior profiles.

We also hypothesized that lower accessibility to resources for health (i.e., placing in a lower environmental quartile/quintile for food, exercise, or SES) would be associated with engaging in health compromising behaviors. Although significance levels should be considered, (*p =* 0.071) results directionally indicated that being in the median county level socioeconomic class (i.e., Yost Quintile 3) and separately being in quartile 1 and 2 of the exercise environment index (i.e., the lowest access) was associated with a greater likelihood of being in health behavior Class 2. Those in the median level Yost quintile may be undergoing something of a paradox, where the wealthier socioeconomic regions are supported by their environment and independent resources, and less wealthy socioeconomic counties are supported by governmental assistance programs, whereas the median county-level socioeconomic classes may represent competency exploitation in a post-COVID-19 pandemic (Simet, 2020). For the exercise environment, this corroborates, as Class 2 is categorized by lower odds of meeting weekly exercise guidelines, meaning their lack of exercise may be a direct result of their county-level environment. This presents valuable insight that the built environment may be driving some cancer-risk associated lifestyles such as inactivity and obesity presented in Class 2.

In the moderated models, being less educated moderated the relationships between the Yost index (area-based SES) and health behavior class. Here, we see that for those who are in the highest county-level socioeconomic grouping (Yost quintile 1), being less educated was associated with a greater likelihood of being in health behavior Class 1, dominated by patterns of binge drinking, being relatively (compared to Class 2) more likely to meet physical activity guidelines, but being relatively more sedentary (compared to Class 2). Here, it may be that coupled with decreased educational attainment, those who fall into Class 1 with lower education (a measure of individual level SES), may be less equipped to interact with living in a higher SES county (Collins, 2016). Accessible psychoeducation resources such as digital health interventions may be an optimal tool for reaching individuals who are more inclined to engage in substances [i.e., binge drinking and tobacco use (cigarette/e-cigarette use)] (Hampton et al., 2024).

In addition, living in a metropolitan area moderated the relationship between exercise environment and health behavior class. Here, we see that for those in a high exercise environment and living in a rural area have a higher likelihood of being in health behavior Class 2, dominated by patterns of low physical activity, difficulty maintaining a healthy weight, and being sedentary. While surprising, individuals considered to have adequate access to exercise opportunities in a rural area are defined as residing in a census block that is within three miles of a recreation facility, whereas adequate access in an urban area is defined as within one mile (County Health Rankings, 2025). For those in rural areas, three miles may seem like a daunting task and not be perceived as accessible. Future research should explore perceptions of adequate access among these rural populations. Moreover, while rural areas tend to have more open space (which can be used for physical activity) research shows that access to natural spaces is associated with less physical activity in rural spaces (Abildso et al., 2021).

Lastly, education showed a significant interaction between the exercise environment and health behavior class in that being more highly educated in a more accessible exercise environment (i.e., Exercise Quintile 5) was associated with being in Class 2. Interestingly, Class 2 is characterized by not meeting sufficient weekly physical activity levels. Here, we may see that being more highly educated reflects having a white-collar job and does not permit enough time to exercise throughout the week. Implementing work-based breaks that promote physical activity throughout the day may be helpful to guide people who fall into this health behavior profile into a more active lifestyle. Along with targeted intervention, however, workplace policy efforts may also need to be considered, as U.S. social norms do not typically prioritize physical activity and well-being (Bramante et al., 2018). More research in this area could benefit the well-being of those who are well-resourced (as reflected in the study findings) yet still struggle to meet health behavior recommendations to mitigate cancer risk.

Taken together, our findings can be used to inform change, grounded in the Built Environment Change (BEC) framework, where real life-built environment features and health behavior associations are detected, affordance moderates the exposure, and the exposure is associated with health issues (i.e., cancer) (Berke & Vernez-Moudon, 2014). Our findings may be able to support change within low activity conducive spaces and allow for policy change within the built environment. Here, environmental change (e.g., implementing more green space, parks, or recreation facilities) can promote physical activity for people who live in these low-access-to-physical-activity environments. Furthermore, in an era with high accessibility to the internet, many emerging adults have access to free online physical activity guides and education. Perhaps, coupled with BEC, psychoeducation (e.g., digital health interventions) would also be an appropriate response to these findings to promote engagement with free, realistic, low-effort physical activity opportunities to promote navigation and engagement with existing information and behavioral tendencies (e.g., social media use). Emerging adults already seek health information independently (Stifjell et al., 2025), but misvalue information based on appearance or instinct (Lim et al., 2022). This presents an opportunity to guide emerging adults in their online health-seeking behavior, and direct them towards accurate, evidence-based tools. Future research should also consider implementing work-based breaks that focus on getting individuals up and moving, which may be beneficial for those in median area-based SES areas where majority of their day is dominated by being at work (Bramante et al., 2018).

Several limitations should be considered when interpreting these findings. The study utilized a public health dataset (i.e., HINTS 2022 Data Linkage Project), meaning variables were not customizable to the study objective. For example, insights for county size are not available within the dataset. We also recognize that health behaviors included in this study (i.e., obesity, binge drinking, physical inactivity, sedentary behavior, inadequate sleep, and smoking/e-cigarette use) are not an exhaustive representation of increased cancer risk among U.S. emerging adults and also that certain health behaviors are worth considering at varying levels (e.g., light/occasional drinking, heavy drinking, and binge drinking are all relevant to cancer risk) (Jun et al., 2023). Further, the cross-sectional nature of these data prohibits causal inference. We are unable to understand how health behaviors evolve, change over time, or detect daily level fluctuations of within-person systems (affect, microsystem, mesosystem) or external factors (weather, season). Future research would benefit from a longitudinal approach among research in this area, as day-to-day lives vary drastically. Also, built environments are extremely diverse across the United States culturally, economically, geographically, and in population density (Arcury et al., 2005; Gopalkrishnan, 2018). Cultural and societal norms may also influence health behaviors within regions. While county-level variables such as Yost index scores and built environment accessibility offer valuable insights, they may not fully capture the lived experiences of the day-to-day lives of emerging adults (Kwan, 2012), as emerging adults are likely geographically mobile and spend time outside of their residential neighborhoods (Wray et al., 2023). This, however, highlights opportunities for further research to utilize methods such as GPS (global positioning system) and ecological momentary assessment (EMA) surveys to understand the lived environment’s influence on health behaviors. Lastly, the present data may underrepresent certain subpopulations of emerging adults, such as those experiencing homelessness or those in temporary living situations.

## Conclusion

This study underscores the importance of acknowledging the BE and SES conditions on health behaviors that have been shown to be associated with increased risk for cancer (Gomez et al., 2015; Wray & Minaker, 2019). With the growing incidence of emerging adult cancers (Bleyer, 2023), it is necessary to conduct further research on these relationships to find optimal methods of promoting health behaviors through the built and socioeconomic environment and provide support to emerging adults who may be negatively impacted by their environments. Alongside the added stressors of navigating major life transitions and novel independence during emerging adulthood may require special attention in utilizing their environments to engage in health-promoting behaviors and promote healthy lifestyles into adulthood.

Our findings highlight both the gaps and opportunities for future research utilizing longitudinal, ecological momentary assessment and/or geospatial methods, and digital health and health interventions. Given our findings of the socioeconomic and built environment on health behaviors, these may be prime locations for in the moment behavior change. The results of this study may also offer valuable implications for individuals in taking advantage of existing resources to overcome deficits in their immediate environment. Further research would benefit from taking a closer look at particularly disadvantaged populations discussed in these findings to find optimal ways of mitigating engagement of cancer-risk associated health behaviors.

## Data Availability

The data that support the study findings are publicly available and released by the National Cancer Institute Health Information National Trends Survey. The study utilized the data from HINTS Cycle 6 (2022): https://hints.cancer.gov/data/download-data.aspx.
